# Diabetes mellitus, systemic inflammation and overactive bladder

**DOI:** 10.3389/fendo.2024.1386639

**Published:** 2024-04-29

**Authors:** Qingliu He, Lizhen Wu, Changqi Deng, Jiatai He, Jun Wen, Chengcheng Wei, Zhijiao You

**Affiliations:** ^1^ Department of Urology, Jinjiang Municipal Hospital, Quanzhou, Fujian, China; ^2^ Department of Urology, The Second Affiliated Hospital of Fujian Medical University, Quanzhou, China; ^3^ Department of Urology, Union Hospital, Tongji Medical College, Huazhong University of Science and Technology, Wuhan, Hubei, China; ^4^ Department of Endocrinology and Metabolism, The Second Affiliated Hospital of Fujian Medical University, Quanzhou, Fujian, China; ^5^ Department of Respiratory and Critical Care Medicine, The First Affiliated Hospital of Chongqing Medical University, Chongqing Medical University, Chongqing, China; ^6^ Department of Urology, The First Affiliated Hospital of Chongqing Medical University, Chongqing, China

**Keywords:** diabetes, inflammation, overactive bladder, NHANES, epidemiology

## Abstract

**Background:**

Increasing evidence emphasizes the potential relationship between diabetes and OAB (overactive bladder). However, large population epidemiology is still lacking.

**Methods:**

This cross-sectional study included six cycle NHANES surveys, with a total of 23863 participants. Logistic regression models were constructed to analyze the association between diabetes mellitus, diabetes-related markers, and inflammatory biomarkers with OAB. Restricted cubic splines were used to analyze the non-linear associations. Mediating analysis was performed to test the effect of inflammatory biomarkers on the relationship between diabetes-related markers and OAB. Finally, machine learning models were applied to predict the relative importance and construct the best-fit model.

**Results:**

Diabetes mellitus participants’ OAB prevalence increased by 77% compared with non-diabetes. As the quartiles of diabetes-related markers increased, the odds of OAB monotonically increased in three models (all p for trend < 0.001). Glycohemoglobin exhibited a linear association with OAB (p for nonlinearity > 0.05). White blood cells significantly mediated the associations between diabetes-related markers (glycohemoglobin, fasting glucose, and insulin) with OAB, and the proportions were 7.23%, 8.08%, and 17.74%, respectively (all p < 0.0001). Neutrophils partly mediated the correlation between (glycohemoglobin, fasting glucose, and insulin) and OAB at 6.58%, 9.64%, and 17.93%, respectively (all p < 0.0001). Machine learning of the XGBoost model constructs the best fit model, and XGBoost predicts glycohemoglobin is the most important indicator on OAB.

**Conclusion:**

Our research revealed diabetes mellitus and diabetes-related markers were remarkably associated with OAB, and systemic inflammation was an important mediator of this association.

## 1 Introduction

Overactive bladder (OAB) is a syndrome characterized by urinary urgency, often accompanied by frequency and nocturia, and may or may not be accompanied by urgent urinary incontinence, which negatively affects the quality of life of patients and their interactions with society (1, 2). There are several theories regarding the pathophysiology of OAB, including the myogenic hypothesis, the urotheliogenic hypothesis, the supraspinal hypothesis, metabolic syndrome, and urinary microbiota involvement, but a full explanation is still missing (3).

As one of the main components of the metabolic syndrome, diabetes mellitus (DM) has profound effects on multiple organ systems, including the kidney and bladder (4, 5). Diabetic cystopathy, also known as diabetic bladder dysfunction (DBD), is a common urological complication whose modern definition encompasses overactive bladder, voiding dysfunction, and urinary retention (6). Some studies estimated that the prevalence rate of diabetic cystopathy was highly varied, ranging from 25% to 90% (7, 8). Several epidemiological studies have shown that OAB is more common in patients with type 2 DM than in the general population, including women with DM treated with insulin and diabetic children aged 11–17 (9–11). Chiu A.F. et al. reported that higher glycosylated hemoglobin levels were independent predictors of OAB symptoms in DM patients, and a study of 36,560 OAB patients in the US found that patients with DM are more persistent and adherent to OAB medications (12, 13). Although it is known that overactive bladder is more common in patients with diabetes, the mechanism by which type 2 diabetes may lead to its development is still unclear (14).

Recent studies have shown that the vicious cycle of chronic inflammation and related stresses is associated with several diabetic complications, including atherosclerosis, nephropathy, and cystopathy, which have been demonstrated in both human and animal experiments (15–17). The pathophysiology of OAB is not well understood; however, chronic systemic inflammation and bladder urothelial inflammation, including some inflammatory proteins and cytokines, may contribute to the onset of OAB (15, 18). An increasing number of studies suggest that DBD and OAB are slow-onset and slow-progressing complications, and among them, inflammatory factors such as IL-1β and NLRP3 play an important role (19, 20). Collectively, based on the importance of systemic inflammation for diabetes mellitus and OAB, respectively, we hypothesized that diabetes mellitus may increase OAB risk by promoting systemic inflammation.

Hence, we conducted a cross-sectional study to investigate the associations of diabetes mellitus with OAB risk based on the National Health and Nutrition Examination Survey (NHANES) 2007–2018. Further, we measured systemic inflammation status from multiple perspectives and explored the mediated effects of systemic inflammation.

## 2 Methods

### 2.1 Study population and design

**Figure 1:**
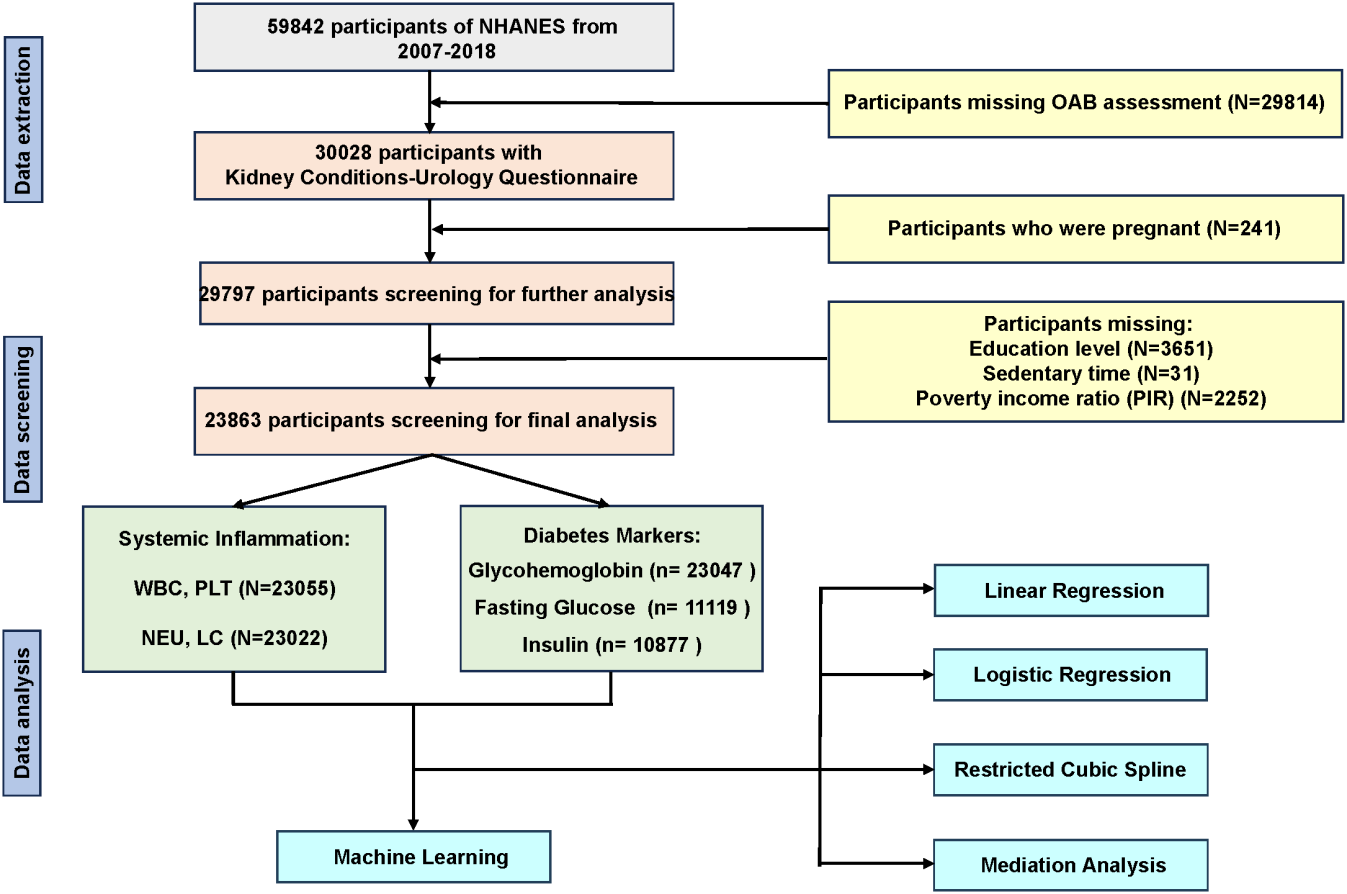
Flowchart of participants selection.

In order to evaluate the health and nutritional status of adults and children in the United States, the National Center for Health Statistics (NCHS) and the Centers for Disease Control and Prevention (CDC) conduct the NHANES research program. Data from the NHANES survey was accessible to the general public via the official website (https://www.cdc.gov/nchs/nhanes/about_nhanes.htm). The NHANES methods were approved by the Centers for Disease Control and Prevention’s National Center for Health Statistics, and informed permission was given by each participant. Cross-sectional data from six consecutive NHANES iterations, conducted between 2007 and 2018, were included into our study. Five iterations in a row with 59842 participants. Initially, 29814 people were removed from the sample as a whole because their OAB assessments were lacking. Next, we removed the individuals (n = 241) who were pregnant from the NHANES questionnaire data. Finally, we eliminated the individuals who lacked information on their poverty income ratio (n = 2252), sedentary time (n = 31), and education level (n = 3651). A total of 23863 individuals were kept for further analysis after these patients were eliminated (**Figure 1**).

### 2.2 Diabetes mellitus and diabetes related markers

(1) Fasting plasma glucose levels between 6.1 and 6.9 mmol/L (2); glycohemoglobin (HbA1c) levels between 6.0% and 6.4%; and (3) self-reported physician diagnosis of prediabetes are the diagnostic criteria for borderline diabetes (prediabetes). Diabetes mellitus is defined as follows (1): plasma glucose level of 7.0 mmol/L while fasting (2); blood glucose level of 11.11 mmol/L after a 2-hour oral glucose tolerance test (3); glycohemoglobin (HbA1c) of 6.5%; and (4) self-reported medical diagnosis of diabetes (21). Serum insulin, fasting glucose, and glycohemoglobin were among the diabetes-related indicators that were derived from NHANES laboratory data.

### 2.3 Overactive bladder symptoms assessment

We assessed overactive bladder symptoms in the NHANES population using the NHANES Kidney Conditions-Urology questionnaire, which covered urge urine incontinence and nocturia, based on the Overactive Bladder Symptom Score [OABSS], which was presented in **Supplementary Table S2** (22). The person who had a total score of ≥3 was determined to have overactive bladder disorder.

### 2.4 Covariates

Covariates were gathered via questionnaire, laboratory, and demographic data. Using a standardized questionnaire, we have incorporated variables such as age, sex, educational attainment, race, poverty income ratio (PIR), alcohol intake, smoking status, sedentary time, hypertension, congestive heart failure, coronary heart disease, angina, heart attack, and stroke history. The NHANES website provides descriptions of each of these variables. Three categories were used to categorize educational levels: below high school, high school, and above high school. A person was considered a smoker if they had smoked 100 cigarettes or more in their lifetime. Anyone who drank alcohol in excess of 12 times in a single year was considered an alcohol user. Weight in kilograms (kg) divided by height in meters squared (m^2^) yielded the BMI. Through participant interviews, the amount of time spent in sedentary activity on a normal day was determined. Through laboratory examination, the number of white blood cells, neutrophils, lymphocytes, and platelets in blood samples were determined. We also identified the diabetes controlling status from “DIQ280 - What was your last A1C level and DIQ290 What does Dr say A1C should be”. If DIQ280 higher than DIQ290, we identified diabetes controlling status was not good.

### 2.5 Statistical analysis

The complex sampling methodology of the NHANES meant that sample weights, clustering, and stratification were all taken into consideration in the data analysis. Using multivariable logistic regression models, we assessed the associations between diabetes, diabetes-related indicators, inflammatory markers, and overactive bladder. Model 1 was not adjusted. Model 2 was adjusted for age, sex, race, education level, poverty income ratio. Model 3 was adjusted for age, sex, race, educational level, poverty income ratio, BMI, smoking, alcohol use, sedentary time, hypertension, congestive heart failure, coronary heart failure, angina, heart attack, stroke. Restricted cubic splines were used to analyze the non-linear associations. Linear regression models were performed to analyze the relationship of diabetes related markers and inflammatory biomarkers. The association between inflammatory biomarkers and diabetes-related indicators was examined using linear regression models. For causal mediation studies, we estimated the direct effect (DE), indirect effect (IE), and total effect (TE) using the R package called “mediation” (23). There should be a connection between the mediator and the result and the exposure (24). The OAB prediction model was constructed using Python software (version 3.7). The xgboost package, adaboost package, lightgbm package, decision tree package, and MLP package execute different machine learning algorithms while modeling different machine learning methods. Subgroup analysis was conducted to analyze the prevalence differ socioeconomics including age, gender, races, education and poverty income ratio (PIR). Last, we conducted the sensitivity analysis using multiple interpolation to supplement the socioeconomic status. We have multiple interpolated the socioeconomic status including poverty income ratio (n = 2252), sedentary time (n = 31), and education level (n = 3651) and to validate the research results. All statistical analyses were performed using R software (version 4.0.1). Significance was set at p < 0.05 (two-sided).

## 3 Results

### 3.1 Population characteristics

**Table 1** summarizes the detailed baseline characteristics of participants by OAB, and **Supplementary Table S1** illustrates the participants with survey-weighted descriptive statistics. A total of 23,863 participants were included in our research, with a median age of 49.7 ± 17.6 years. Among them, 4894 participants were OAB participants, and 18969 were non-OAB participants. We found inflammatory markers, including white blood cells, neutrophil count, and platelet count, had different distributions between OAB participants and non-OAB participants. OAB participants had a higher white blood cell count and neutrophil count. Moreover, OAB participants had higher levels of diabetes-related markers, including glycohemoglobin, fasting glucose, and insulin.

**Table 1:** Characteristics of participants by OAB: NHANES 2007–2018.

Variables	Overall	OAB participants (n = 4894)	Non-OAB participants (n =18969)	P Value
**Age, years**	49.7 ± 17.6	60.0 ± 15.5	47.0 ± 17.2	<0.001
Gender, %				<0.001
Male	11880 (49.7%)	2027 (41.4%)	9840 (51.9%)	
Female	12009 (50.3%)	2867 (58.6%)	9129 (48.1%)	
Education, %				<0.001
Below high school	5663 (23.7%)	1730 (35.3%)	3920 (20.7%)	
High school	5467 (22.9%)	1164 (23.8%)	4303 (22.7%)	
Above high school	12759 (53.4%)	2000 (40.9%)	10746 (56.6%)	
Race/ethnicity, %				<0.001
Non-Hispanic White	10518 (44.0%)	2009 (41.1%)	8509 (44.8%)	
Non-Hispanic Black	4974 (20.8%)	1378 (28.2%)	3583 (18.9%)	
Other Hispanic	2417 (10.1%)	513 (10.5%)	1904 (10.0%)	
Mexican American	3474 (14.5%)	683 (14.0%)	2791 (14.7%)	
Others race	2506 (10.5%)	311 (6.4%)	2182 (11.6%)	
**Body mass index, kg/m^2^**	29.3 ± 7.0	31.2 ± 7.9	28.8 ± 6.7	<0.001
**Poverty income ratio (PIR)**	2.5 ± 1.6	2.1 ± 1.5	2.6 ± 1.6	<0.001
**Smoking, %**	10835 (45.4%)	2528 (51.7%)	8307 (43.7%)	<0.001
**Alcohol use, %**	17507 (73.3%)	3302 (67.5%)	14205 (74.8%)	<0.001
Sedentary time, hrs				0.048
<3 h	6417 (26.9%)	1254 (25.6%)	5150 (27.2%)	
3–6 h	8415 (35.2%)	1783 (36.4%)	6629 (34.9%)	
>6 h	9057 (37.9%)	1857 (37.9%)	7200 (37.9%)	
**Hypertension, %**	8791 (36.8%)	2849 (58.2%)	5942 (31.3%)	<0.001
**Congestive heart failure, %**	719 (3.0%)	328 (6.7%)	391 (2.1%)	<0.001
**Coronary heart disease, %**	917 (3.8%)	342 (7.0%)	575 (3.0%)	<0.001
**Angina, %**	569 (2.4%)	251 (5.1%)	318 (1.7%)	<0.001
**Heart attack, %**	941 (3.9%)	372 (7.6%)	569 (3.0%)	<0.001
**Stroke, %**	831 (3.5%)	358 (7.3%)	473 (2.5%)	<0.001
**Diabetes, %**	3119 (13.1%)	1282 (26.2%)	1837 (9.7%)	<0.001
Inflammatory markers				
White blood cell count, 1000 cells/uL	7.2 ± 3.6	7.4 ± 3.5	7.2 ± 3.7	<0.001
Neutrophils count, 1000 cell/u	4.3 ± 1.8	4.4 ± 2.1	4.2 ± 1.7	<0.001
Lymphocyte count, 1000 cell/uL	2.2 ± 2.7	2.2 ± 2.3	2.2 ± 2.8	0.654
Platelet count, 1000 cells/uL	243.4 ± 64.7	242.8 ± 74.0	243.6 ± 62.1	0.002
Diabetes related markers				
Glycohemoglobin, %	5.8 ± 1.1	6.2 ± 1.4	5.7 ± 1.0	<0.001
Fasting Glucose, mmol/L	6.1 ± 2.0	6.7 ± 2.6	6.0 ± 1.8	<0.001
Insulin, uU/mL	13.9 ± 17.8	15.6 ± 19.0	13.4 ± 17.5	<0.001

Data are presented as median (IQR) or N (%). If it is a continuous variable, the Kruskal–Wallis rank sum test was used to determine it. The p-value for continuous variables with a theoretical value of <10 was determined using Fisher’s exact probability test. With regard to categorical data, the p-value was calculated using weighted chi-square. Values are presented as means + SD or %. SD, standards deviation.

### 3.2 Association between diabetes mellitus and overactive bladder

**Figure 2:**
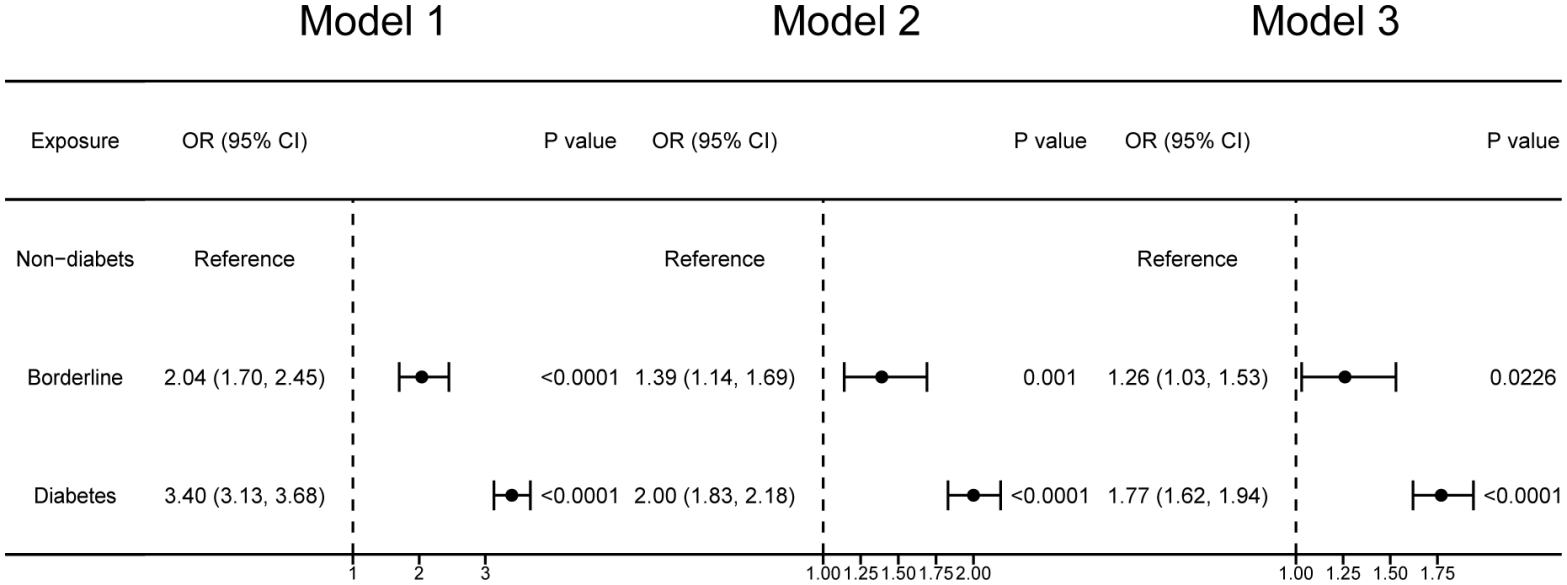
Forest plots illustrating the correlation between diabetes mellitus and overactive bladder. Model 1 was not adjusted. Model 2 was adjusted for age, sex, race, education level, poverty income ratio. Model 3 was adjusted for age, sex, race, educational level, poverty income ratio, BMI, smoking, alcohol use, sedentary time, hypertension, congestive heart failure, coronary heart failure, angina, heart attack, stroke.

**Figure 2** reveals the association between diabetes mellitus and OAB. We identified diabetes mellitus as no-diabetes, borderline, and diabetes. Compared with non-diabetes participants, borderline diabetes participants’ OAB prevalence increased by 26% after adjusting for all covariates (Model 3: OR = 1.26; 95% CI: 1.03, 1.53). Diabetes mellitus participants’ OAB prevalence increased 77% compared with non-diabetes participants in the full adjusted model (Model 3: OR = 1.77; 95% CI: 1.62, 1.94).

### 3.3 Association between diabetes related markers and overactive bladder

**Figure 3:**
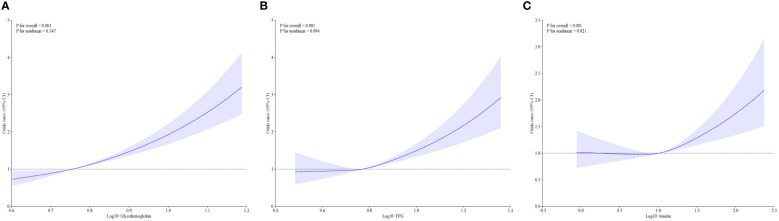
The non-linear associations between diabetes related markers and overactive bladder by restricted cubic splines. **(A)** The association between glycohemoglobin and overactive bladder; **(B)** The association fasting glucose and overactive bladder; **(C)** The association insulin and overactive bladder; Model was adjusted for age, sex, race, educational level, poverty income ratio, BMI, smoking, alcohol use, sedentary time, hypertension, congestive heart failure, coronary heart failure, angina, heart attack, stroke.

**Table 2** shows the associations of diabetes-related markers with OAB in adults using multiple logistic regression analysis. We found the log10-transformed exposures, including glycohemoglobin, fasting glucose, and insulin, had a significant positive association with OAB. As the quartiles of diabetes-related markers increased, the odds of OAB monotonically increased in three models (all p for trend <0.01). In addition, the cubic spline curves revealed non-linear associations between diabetes-related markers and overactive bladder (**Figure 3**). Glycohemoglobin exhibited a linear association with OAB (p for nonlinearity >0.05), while associations between fasting glucose, insulin, and OAB were nonlinearity curves (p for nonlinearity < 0.05).

**Table 2:** Multiple logistic regression associations of diabetes related markers with overactive bladder in adults.

Exposure	Model	Continuous log10- transformed Exposures	Quartile 1	Quartile 2	Quartile 3	Quartile 4	p for trend
			OR	OR (95%CI)	OR (95%CI)	OR (95%CI)	
Glycohemoglobin (n= 23047)	Model 1	424.10 (273.73, 657.08) ***	1.00 (Ref.)	1.29 (1.14, 1.46) ***	2.11 (1.88, 2.36) ***	4.06 (3.64, 4.54) ***	<0.001
	Model 2	21.33 (13.14, 34.63) ***	1.00 (Ref.)	1.01 (0.88, 1.15)	1.15 (1.02, 1.30) *	1.60 (1.41, 1.81) ***	<0.001
	Model 3	13.37 (8.16, 21.91) ***	1.00 (Ref.)	1.01 (0.88, 1.15)	1.14 (1.00, 1.29) *	1.46 (1.29, 1.66) ***	<0.001
Fasting Glucose (n= 11119)	Model 1	25.32 (16.93, 37.87) ***	1.00 (Ref.)	1.10 (0.95, 1.27)	1.42 (1.24, 1.64) ***	2.60 (2.28, 2.97) ***	<0.001
	Model 2	5.98 (3.83, 9.33) ***	1.00 (Ref.)	1.00 (0.86, 1.17)	1.08 (0.93, 1.26)	1.49 (1.28, 1.72) ***	<0.001
	Model 3	4.36 (2.77, 6.86) ***	1.00 (Ref.)	1.00 (0.85, 1.17)	1.03 (0.88, 1.21)	1.33 (1.14, 1.55) ***	<0.001
Insulin (n= 10877)	Model 1	1.66 (1.44, 1.92) ***	1.00 (Ref.)	1.08 (0.94, 1.24)	1.30 (1.14, 1.49) ***	1.51 (1.32, 1.72) ***	<0.001
	Model 2	1.56 (1.34, 1.83) ***	1.00 (Ref.)	1.01 (0.87, 1.17)	1.17 (1.01, 1.36) *	1.41 (1.23, 1.63) ***	<0.001
	Model 3	1.37 (1.16, 1.60) ***	1.00 (Ref.)	1.00 (0.86, 1.16)	1.13 (0.97, 1.31)	1.27 (1.09, 1.47) **	<0.001

Model 1 was not adjusted.

Model 2 was adjusted for age, sex, race, education level, poverty income ratio.

Model 3 was adjusted for age, sex, race, educational level, poverty income ratio, BMI, smoking, alcohol use, sedentary time, hypertension, congestive heart failure, coronary heart failure, angina, heart attack, stroke.

OR, odds ratio; CI, confidence interval; Ref., reference; *p < 0.05, **p < 0.01 and ***p < 0.001.

### 3.4 Association between inflammatory markers and overactive bladder

As shown in **Table 3**, we found significant correlations between inflammatory markers (white blood cell count, neutrophil count, lymphocyte count, and platelet count) and OAB. In the multiple linear regression analysis, we found log10-transformed white blood cell count and neutrophil count were significantly associated with OAB (all p < 0.001). As the quartiles of white blood cell count and neutrophil count increased, the odds of OAB monotonically increased in three models (p for trend <0.001). Restricted cubic splines reveal the non-linear associations between inflammatory markers and OAB. Four inflammatory markers exhibited a non-linear association with OAB with a statistical difference (**Supplementary Figure S1**).

**Table 3:** Multiple linear regression associations of inflammatory markers with overactive bladder in adults.

Exposure	Model	Continuous log10- transformed Exposures	Quartile 1	Quartile 2	Quartile 3	Quartile 4	p for trend
			OR	OR (95%CI)	OR (95%CI)	OR (95%CI)	
WBC count (n= 23055)	Model 1	1.96 (1.52, 2.52) ***	1.00 (Ref.)	1.02 (0.93, 1.12)	1.10 (1.00, 1.20) *	1.20 (1.10, 1.32) ***	P < 0.001
	Model 2	3.34 (2.54, 4.41) ***	1.00 (Ref.)	1.09 (0.99, 1.21)	1.25 (1.13, 1.38) ***	1.46 (1.32, 1.61) ***	P < 0.001
	Model 3	2.19 (1.65, 2.90) ***	1.00 (Ref.)	1.05 (0.95, 1.16)	1.15 (1.04, 1.27) **	1.26 (1.14, 1.40) ***	P < 0.001
Neutrophil count (n= 23020)	Model 1	1.76 (1.45, 2.12) ***	1.00 (Ref.)	1.01 (0.92, 1.11)	1.12 (1.02, 1.22) *	1.26 (1.15, 1.38) ***	P < 0.001
	Model 2	2.60 (2.10, 3.21) ***	1.00 (Ref.)	1.11 (1.00, 1.23) *	1.24 (1.12, 1.37) ***	1.50 (1.35, 1.66) ***	P < 0.001
	Model 3	1.88 (1.52, 2.34) ***	1.00 (Ref.)	1.06 (0.96, 1.18)	1.14 (1.03, 1.26) *	1.30 (1.17, 1.44) ***	P < 0.001
Lymphocyte count (n= 23020)	Model 1	0.63 (0.51, 0.78) ***	1.00 (Ref.)	0.78 (0.71, 0.86) ***	0.68 (0.62, 0.74) ***	0.84 (0.77, 0.92) ***	P < 0.001
	Model 2	1.16 (0.93, 1.45)	1.00 (Ref.)	0.94 (0.84, 1.04)	0.87 (0.79, 0.95) **	1.08 (0.98, 1.18)	P < 0.001
	Model 3	1.02 (0.81, 1.28)	1.00 (Ref.)	0.94 (0.84, 1.04)	0.85 (0.77, 0.94) **	1.01 (0.91, 1.11)	P < 0.001
Platelet count (n= 23055)	Model 1	0.61 (0.47, 0.80) ***	1.00 (Ref.)	0.78 (0.71, 0.86) ***	0.72 (0.66, 0.79) ***	0.89 (0.82, 0.97) *	P < 0.001
	Model 2	1.26 (0.94, 1.70)	1.00 (Ref.)	0.93 (0.85, 1.03)	0.92 (0.83, 1.02)	1.11 (1.00, 1.22) *	P < 0.001
	Model 3	1.28 (0.95, 1.73)	1.00 (Ref.)	0.95 (0.86, 1.05)	0.94 (0.85, 1.04)	1.10 (1.00, 1.22)	P < 0.001

Model 1 was not adjusted.

Model 2 was adjusted for age, sex, race, education level, poverty income ratio.

Model 3 was adjusted for age, sex, race, educational level, poverty income ratio, BMI, smoking, alcohol use, sedentary time, hypertension, congestive heart failure, coronary heart failure, angina, heart attack, stroke and diabetes.

OR, odds ratio; CI, confidence interval; Ref., reference; *p < 0.05, **p < 0.01 and ***p < 0.001.

### 3.5 Association between diabetes related markers and inflammatory markers

From the multiple linear regression analysis, we found a significant correlation between the diabetes-related markers (glycohemoglobin, fasting glucose, and insulin) and inflammatory markers (white blood cells, neutrophils, lymphocytes, and platelets), which is shown in **Table 4**. Diabetes-related markers all showed a positive association with inflammatory markers, with statistical differences. Among them, we found glycohemoglobin to be the most significantly correlated with inflammatory markers. Glycohemoglobin was linked to higher WBC (β = 4.67; 95% CI: 3.76, 5.58), neutrophil (β = 2.56; 95% CI: 2.11, 3.01), lymphocyte (β = 1.80; 95% CI: 1.11, 2.49), and platelet counts (β = 105.68; 95% CI: 89.89, 121.47).

**Table 4:** Multiple linear regression associations of inflammatory markers with diabetes related markers in adults.

Outcome	Glycohemoglobin β (95%CI)	p-value	Fasting Glucose β (95%CI)	p-value	Insulin β (95%CI)	p-value
WBC count	4.67 (3.76, 5.58)	<0.001	2.18 (1.62, 2.73)	<0.001	1.05 (0.90, 1.20)	<0.001
Neutrophil count	2.56 (2.11, 3.01)	<0.001	1.65 (1.27, 2.03)	<0.001	0.69 (0.59, 0.79)	<0.001
Lymphocyte count	1.80 (1.11, 2.49)	<0.001	0.48 (0.16, 0.79)	0.003	0.28 (0.20, 0.37)	<0.001
Platelet count	105.68 (89.89, 121.47)	<0.001	32.68 (18.58, 46.78)	<0.001	11.29 (7.54, 15.05)	<0.001

Model was adjusted for age, sex, race, educational level, poverty income ratio, smoking, BMI, alcohol use, sedentary time, hypertension, congestive heart failure, coronary heart failure, angina, heart attack, stroke. WBC, white blood cell; CI, confidence interval.

### 3.6 Systemic inflammation mediated the association between diabetes and overactive bladder

**Figure 4:**
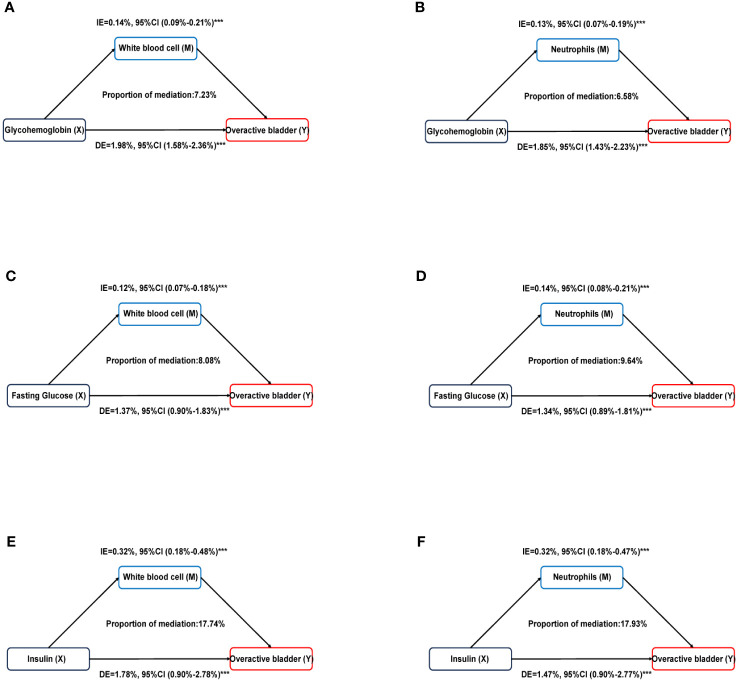
Estimated proportion of the association between diabetes related markers and overactive bladder mediated by inflammatory markers. **(A)** Glycohemoglobin mediated by White blood cell; **(B)** Glycohemoglobin mediated by Neutrophils; **(C)** Fasting glucose mediated by White blood cell; **(D)** Fasting glucose mediated by Neutrophils; **(E)** Insulin mediated by White blood cell; **(F)** Insulin mediated by Neutrophils. Models were adjusted for age, sex, race, educational level, poverty income ratio, BMI, smoking, alcohol use, sedentary time, hypertension, congestive heart failure, coronary heart failure, angina, heart attack, stroke. IE, indirect effect; DE, direct effect; Proportion of mediation = IE / (DE + IE); ***P < 0.001.

Mediation analysis was used to evaluate the mediation effect between the diabetes-related markers and OAB by systemic inflammation (**Supplementary Table S3**). We found the white blood cells significantly mediated the associations between diabetes-related markers (glycohemoglobin, fasting glucose, and insulin) and OAB, and the proportions were 7.23%, 8.08%, and 17.74%, respectively. (all p < 0.0001). Similarly, neutrophils partly mediated the correlation between glycohemoglobin, fasting glucose, and insulin with OAB at 6.58%, 9.64%, and 17.93%, respectively (all p < 0.0001) (**Figure 4**). There were no significant mediation effects observed in the associations of diabetes-related markers with overactive bladder by lymphocyte or platelet (all p > 0.05).

### 3.7 Using machine learning predicted diabetes related markers and inflammatory markers with overactive bladder

**Figure 5:**
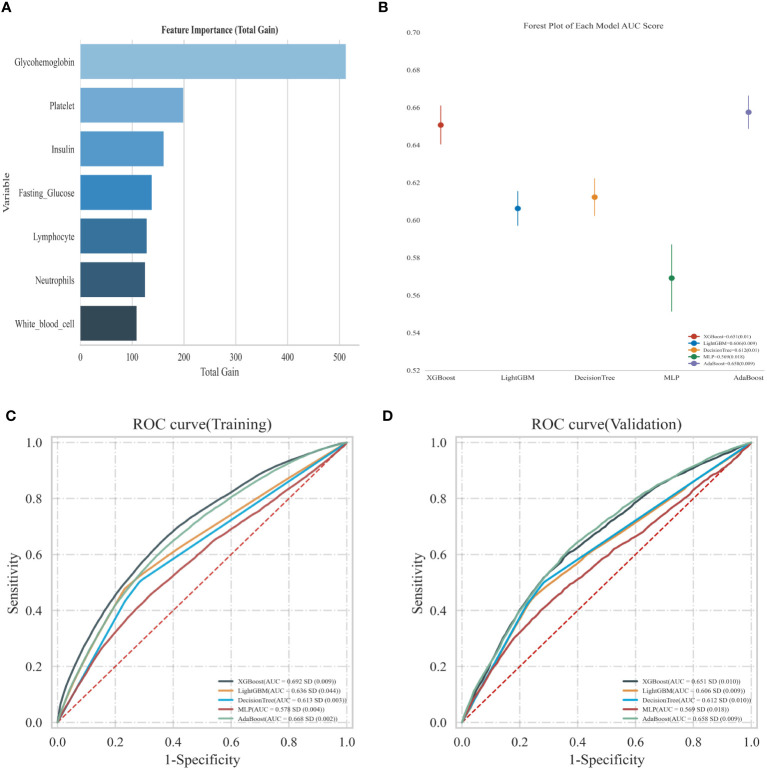
**(A)** Using XGBoost model predict the relative importance; **(B)** Forest map shows the ROC results of OAB prediction by each model; **(C)** Predicted ROC curve of adverse events in the training sets of the five models; **(D)** Predicted ROC curve of adverse events in the validation sets of the five models.

As shown in **Figure 5**, we used machine learning in the XGBoost model to determine the relative significance of the diabetes-related markers and inflammatory markers in OAB. We observed that glycohemoglobin was the most important indicator in determining OAB. The forest chart below shows the ROC results of OAB prediction by each machine learning model, including XGBboost, LightGBM, Decision Tree, MLP, and AdaBoost. The error lines in the chart are the ROC mean value and SD. Among all the current models, XGBboost is the best-performing training set; meanwhile, the best validation set is XGBboost as well (sorted by AUC).

### 3.8 Subgroup analysis and sensitivity analysis

As shown in **Supplementary Table S7**, we conducted subgroup analysis by socioeconomics including age, gender, race, education, ratio of family income to assessed the associations between diabetes and OAB. In the age <60 group, compared with non-diabetes participants, borderline diabetes participants’ OAB prevalence increased by 146%% after adjusting for all covariates and diabetes mellitus participants’ OAB prevalence increased 67%. However, this association was not statistically different in the older population. In addition, it is likely that male, education level more than high school, non-Hispanic Black and middle income (PIR 1.0-3.0) participants had significant significance of diabetes and OAB association. We have multiple interpolated the socioeconomic status including poverty income ratio (n = 2252), sedentary time (n = 31), and education level (n = 3651) and to validate the research results. We found that multiple interpolation results had almost correlated result with delete missing values results. (**Supplementary Tables S4–S6**). In the sensitivity analysis of model 3, we further adjusted the covariates of diabetes controlling status and they had almost same result with previous result.

## 4 Discussion

The presence of OAB exerts a substantial impact on the overall well-being and health status of patients, significantly burdening their physical and mental health as well as their overall quality of life. However, the pathophysiology of OAB is complicated, and the underlying processes remain unknown. This research examines the link between OAB, diabetes, and systemic inflammation, as well as the associated variables that influence bladder overactivity.

First, we investigated the association between diabetes and OAB. The prevalence of OAB in the diabetes group increased by 77% compared to those without diabetes, and multiple logistic regression analysis showed positive correlations between diabetes-related markers and overactive bladder in adults. We also identified inflammatory markers that were significantly correlated with OAB and diabetes-related markers by multiple linear regression. Mediation analysis showed that white blood cells strongly influenced the correlations between diabetes-related indicators (glycohemoglobin, fasting glucose, and insulin) and OAB. Through the application of machine learning, we have finally identified glycoproteins as the most significant markers for bladder overactivity.

Previously, several studies have found that diabetes is an independent risk factor for OAB (12, 25). A number of clinical trials have also shown the connection between OAB and diabetes (26–28), which is consistent with the research results in this paper. On the one hand, urotheliogenic has been identified as the main cause of OAB (29, 30), and the changes in the urothelium may lead to increased spontaneous activity of the detrusor muscle, causing symptoms of OAB to worsen (31). On the other hand, Wang et al. reported that diabetic OAB patients noted lower expression of urothelial adhesion and higher urothelial inflammation (17). Moreover, diabetes is associated with increased levels of systemic inflammatory markers, which have major effects (32, 33). However, the above research is mostly single-center and with a small sample. The evidence of large-population epidemiology is still lacking. Through our research, we found white blood cells and neutrophils mediated the correlation between glucose, fasting glucose, and insulin and an overactive bladder. Therefore, our hypothesis based on the analysis results of the association between OAB, diabetes, and systemic inflammation is that diabetes mellitus may increase OAB risk by promoting systemic inflammation.

The primary advantages of this research are as follows: Firstly, we conducted a cross-sectional study based on the National Health and Nutrition Examination Survey (NHANES) 2007–2018, which includes an unprecedented amount of data. Second, we suggest for the first time that inflammation mediates the relationship between diabetes and OAB. Finally, glycohemoglobin has been regarded as the most significant marker of diabetes-related indicators and inflammatory markers for bladder overactivity, which contains some clinical value. However, our study still has some limitations. First, the data in this paper is cross-sectional; therefore, we are unable to speculate on cause-and-effect relationships. Second, to a certain extent, they had some overlapping diagnosis between OAB and diabetic cystopathy. And, we could not identify the diabetic cystopathy with OAB. According to AUA guide (34), OAB is not a disease, but a complex syndrome. Our research was mainly focused on the syndrome of OAB diagnosis which based on clinical signs and symptoms. Last, NHANES was a descriptive study and could not prove related risk between diabetes and urinary infections or overactive bladder. We could only provide the association cross-sectional relevance between the OAB and diabetes. Our research illustrated that diabetes associated with OAB was partly attributed by systemic inflammation in terms of public epidemiology. Systemic inflammation is different from urinary system inflammation or urinary system infection. It is true that infection or neurogenic lesions may partly lead to OAB as well which need further study.

## 5 Conclusion

Our research is the first large-population epidemiology that revealed that diabetes mellitus and diabetes-related markers were remarkably associated with OAB in the American general adult population. We also observed systemic inflammation as an important mediator between diabetes-related markers and OAB, which will guide the direction for further study on OAB function and mechanism.

## Data availability statement

The original contributions presented in the study are included in the article/**Supplementary Material**. Further inquiries can be directed to the corresponding authors.

## Ethics statement

The studies involving humans were approved by NAHNES Institutional Review Board (IRB)/NCHS Research Ethics Review Board (ERB). The studies were conducted in accordance with the local legislation and institutional requirements. The participants provided their written informed consent to participate in this study.

## Author contributions

QH: Writing – review & editing, Writing – original draft, Visualization, Software, Methodology, Formal analysis, Data curation, Conceptualization. LW: Writing – review & editing, Supervision, Methodology, Funding acquisition, Conceptualization. CD: Writing – review & editing, Methodology, Conceptualization. JH: Writing – review & editing, Methodology, Conceptualization. JW: Writing – review & editing, Validation. CW: Writing – review & editing, Writing – original draft, Visualization, Software, Methodology, Formal analysis, Data curation, Conceptualization. ZY: Writing – review & editing, Supervision, Methodology, Funding acquisition, Conceptualization.
