# Supplementary material for: Diabetes mellitus, systemic inflammation and overactive bladder
Source: Front Endocrinol (Lausanne). 2024 Apr 29;15:1386639. doi: 10.3389/fendo.2024.1386639 (PMC11091467; doi:10.3389/fendo.2024.1386639)
Supplement: Supplementary file 2 [file DataSheet_2.docx]

Table S1. Indicators with their corresponding abbreviations and data fields.

| **Abbreviations** | **Fields** |
| --- | --- |
| RBC | Red blood cell |
| HCT | hematocrit percentage |
| MCV | mean corpuscular volume |
| MCHC | mean corpuscular hemoglobin concentration |
| RDW | Red blood cell (erythrocyte) distribution width |
| RET | Reticulocyte count |
| NRBC | Nucleated red blood cell percentage |
| MRV | Mean reticulocyte volume |
| MSCV | Mean sphered cell volume |
| IRF | Immature reticulocyte fraction |
| WBC | White blood cell |
| BASO | Basophil |
| EO | Eosinophil |
| MONO | monocyte |
| NEUT | Neutrophil |
| LYMPH | lymphocyte |
| MPV | mean platelet volume |
| PDW | Platelet distribution width |
| PLT | Platelet |
| SII | systemic immune-inflammation index |
| PLR | platelet-to-lymphocyte ratio |
| NLR | neutrophil-to-lymphocyte ratio |
| PPN | product of platelet count and neutrophil count |
| FDR | false discovery rate |
| HR | hazard ratio |
| SD | standard deviation. |

Table S2. Stratified analyses of RBC category.

|  |  | MCV |  | MCHC |  | PDW |  |
| --- | --- | --- | --- | --- | --- | --- | --- |
|  | N | HR (95% CI) P-value | p for Interaction | HR (95% CI) P-value | p for Interaction | HR (95% CI) P-value | p for Interaction |
| **Age** |  |  | 0.7724 |  | 0.9436 |  | 0.5865 |
| ≤60 | 287423 | 0.92 (0.86, 1.00) 0.0392 |  | 0.93 (0.86, 1.00) 0.0602 | | 1.07 (1.00, 1.15) 0.0352 | |
| >60 | 179571 | 0.91 (0.86, 0.97) 0.0046 |  | 0.93 (0.87, 0.99) 0.0216 | | 1.10 (1.04, 1.16) 0.0004 | |
| **Gender** |  |  | 0.586 |  | 0.5935 |  | 0.6691 |
| Male | 214089 | 0.89 (0.84, 0.94) 0.0002 |  | 0.91 (0.85, 0.97) 0.0023 | | 1.10 (1.05, 1.16) 0.0002 | |
| Female | 252905 | 0.93 (0.85, 1.00) 0.0627 |  | 0.93 (0.86, 1.01) 0.0942 | | 1.08 (1.02, 1.16) 0.0157 | |
| **Race** |  |  | 0.3555 |  | 0.5289 |  | 0.9057 |
| White European | 441207 | 0.90 (0.85, 0.94) <0.0001 |  | 0.91 (0.86, 0.96) 0.0006 | | 1.10 (1.05, 1.14) <0.0001 | |
| Mixed | 2717 | 0.72 (0.41, 1.28) 0.2656 |  | 0.70 (0.40, 1.24) 0.2227 | | 1.18 (0.44, 3.14) 0.7441 | |
| South Asian | 8798 | 0.98 (0.71, 1.36) 0.9256 |  | 1.00 (0.72, 1.39) 0.9871 | | 1.24 (1.00, 1.54) 0.0537 | |
| Black | 7073 | 1.07 (0.70, 1.64) 0.7513 |  | 1.05 (0.68, 1.64) 0.8156 | | 0.85 (0.50, 1.43) 0.5299 | |
| Others | 7199 | 0.98 (0.65, 1.48) 0.9387 |  | 0.95 (0.64, 1.41) 0.8156 | | 1.05 (0.68, 1.64) 0.8197 | |
| **Smoking status (%)** |  |  | 0.5633 |  | 0.0173 |  | 0.4521 |
| Current | 48996 | 0.87 (0.77, 0.98) 0.0191 |  | 0.83 (0.73, 0.94) 0.0031 | | 1.13 (1.03, 1.25) 0.0111 | |
| Former | 161651 | 0.88 (0.81, 0.95) 0.0007 |  | 0.89 (0.82, 0.96) 0.0027 | | 1.10 (1.03, 1.17) 0.0045 | |
| Never | 254506 | 0.93 (0.87, 1.01) 0.0771 |  | 0.97 (0.90, 1.05) 0.5097 | | 1.08 (1.01, 1.15) 0.0177 | |
| **Alcohol drinker status (%)** | |  | 0.0128 |  | 0.1447 |  | 0.0918 |
| Current | 429274 | 0.90 (0.85, 0.95) <0.0001 |  | 0.91 (0.86, 0.96) 0.0007 | | 1.10 (1.05, 1.15) <0.0001 | |
| Former | 16682 | 0.77 (0.62, 0.95) 0.0152 |  | 0.82 (0.66, 1.03) 0.0933 | | 1.03 (0.86, 1.23) 0.7758 | |
| Never | 20387 | 1.16 (0.90, 1.49) 0.2404 |  | 1.13 (0.88, 1.44) 0.3422 | | 1.16 (1.02, 1.33) 0.0226 | |
| **Qualifications (%)** |  |  | 0.7886 |  | 0.2001 |  | 0.2927 |
| With college or university degree | 315886 | 0.90 (0.85, 0.95) 0.0002 |  | 0.90 (0.85, 0.95) 0.0003 | | 1.11 (1.06, 1.16) <0.0001 | |
| Without college or university degree | 151108 | 0.91 (0.83, 1.00) 0.0624 |  | 0.97 (0.88, 1.07) 0.5396 | | 1.05 (0.95, 1.15) 0.3379 | |
| **Diabetes history** |  |  | 0.8351 |  | 0.2787 |  | 0.1385 |
| Yes | 24210 | 0.87 (0.75, 1.00) 0.0522 |  | 0.97 (0.84, 1.13) 0.6945 | | 1.05 (0.93, 1.20) 0.4226 | |
| No | 441303 | 0.91 (0.86, 0.96) 0.0003 |  | 0.91 (0.86, 0.96) 0.0009 | | 1.10 (1.05, 1.15) <0.0001 | |
| **Hypertension** |  |  | 0.1167 |  | 0.2187 |  | 0.6231 |
| Yes | 111708 | 0.86 (0.79, 0.93) <0.0001 |  | 0.88 (0.81, 0.95) 0.0016 | | 1.08 (1.01, 1.16) 0.0263 | |
| No | 355286 | 0.93 (0.87, 0.99) 0.0178 |  | 0.94 (0.88, 1.00) 0.0470 | | 1.11 (1.05, 1.16) <0.0001 | |
| **BMI** |  |  | 0.9545 |  | 0.9102 |  | 0.9303 |
| ≤25 | 154618 | 0.89 (0.80, 0.99) 0.0256 |  | 0.90 (0.81, 1.00) 0.0534 | | 1.11 (1.02, 1.20) 0.0183 | |
| >25 | 312376 | 0.89 (0.84, 0.94) <0.0001 |  | 0.90 (0.85, 0.96) 0.0006 | | 1.11 (1.06, 1.16) <0.0001 | |

Table S3. Stratified analyses of immature red category.

|  |  | NRBC | | MRV | | MSVC | | | IRF | | |  |
| --- | --- | --- | --- | --- | --- | --- | --- | --- | --- | --- | --- | --- |
|  | N | HR (95% CI) P-value | p for Interaction | HR (95% CI) P-value | p for Interaction | | HR (95% CI) P-value | p for Interaction | | HR (95% CI) P-value | p for Interaction | |
| **Age** |  |  | 0.0842 |  | 0.3621 | |  | 0.6102 | |  | 0.1978 | |
| ≤60 | 287423 | 0.71 (0.48, 1.04) 0.0785 | | 0.99 (0.91, 1.06) 0.7004 | | 0.89 (0.82, 0.96) 0.0031 | | | 1.05 (0.98, 1.14) 0.1755 | | |  |
| >60 | 179571 | 0.93 (0.83, 1.05) 0.2593 | | 0.94 (0.89, 1.00) 0.0557 | | 0.87 (0.81, 0.92) <0.0001 | | | 0.99 (0.93, 1.05) 0.7303 | | |  |
| **Gender** |  |  | 0.6935 |  | 0.852 | |  | 0.8066 | |  | 0.7935 | |
| Male | 214089 | 0.86 (0.73, 1.03) 0.0949 | | 0.94 (0.89, 1.00) 0.0550 | | 0.87 (0.82, 0.93) <0.0001 | | | 1.03 (0.97, 1.09) 0.3903 | | |  |
| Female | 252905 | 0.91 (0.77, 1.06) 0.2287 | | 0.93 (0.86, 1.01) 0.0900 | | 0.88 (0.82, 0.96) 0.0035 | | | 1.01 (0.93, 1.10) 0.7473 | | |  |
| **Race** |  |  | 0.8223 |  | 0.5269 | |  | 0.5163 | |  | 0.4684 | |
| White European | 441207 | 0.90 (0.80, 1.01) 0.0681 | | 0.94 (0.89, 0.98) 0.0091 | | 0.87 (0.83, 0.92) <0.0001 | | | 1.03 (0.98, 1.08) 0.2598 | | |  |
| Mixed | 2717 | 0.09 (0.00, Inf) 0.9962 |  | 0.70 (0.30, 1.62) 0.4040 | | 0.87 (0.40, 1.91) 0.7293 | | | 0.74 (0.32, 1.68) 0.4661 | | |  |
| South Asian | 8798 | 0.06 (0.00, Inf) 0.9945 |  | 1.27 (0.89, 1.82) 0.1947 | | 1.20 (0.81, 1.77) 0.3574 | | | 0.92 (0.60, 1.40) 0.6919 | | |  |
| Black | 7073 | 0.07 (0.00, inf.) 0.9857 |  | 0.97 (0.56, 1.67) 0.9026 | | 1.08 (0.66, 1.75) 0.7684 | | | 1.09 (0.62, 1.91) 0.7768 | | |  |
| Others | 7199 | 0.07 (0.00, inf.) 0.9927 |  | 0.83 (0.52, 1.30) 0.4091 | | 0.81 (0.51, 1.30) 0.3853 | | | 0.67 (0.40, 1.12) 0.1234 | | |  |
| **Smoking status (%)** |  |  | 0.0124 |  | 0.3279 | |  | 0.4991 | |  | 0.4228 | |
| Current | 48996 | 0.05 (0.00, inf.) 0.9892 |  | 1.02 (0.91, 1.16) 0.6902 | | 0.92 (0.82, 1.04) 0.1841 | | | 1.11 (0.97, 1.26) 0.1233 | | |  |
| Former | 161651 | 0.99 (0.89, 1.09) 0.7629 | | 0.94 (0.87, 1.01) 0.0897 | | 0.86 (0.79, 0.93) <0.0001 | | | 1.02 (0.95, 1.10) 0.5779 | | |  |
| Never | 254506 | 0.05 (0.00, inf.) 0.9833 |  | 0.91 (0.85, 0.98) 0.0101 | | 0.87 (0.81, 0.94) 0.0005 | | | 1.00 (0.93, 1.08) 0.9594 | | |  |
| **Alcohol drinker status (%)** | |  | 0.7143 |  | 0.0019 | |  | 0.0035 | |  | 0.2865 | |
| Current | 429274 | 0.90 (0.80, 1.01) 0.0657 | | 0.93 (0.89, 0.98) 0.0057 | | 0.87 (0.82, 0.91) <0.0001 | | | 1.02 (0.97, 1.08) 0.4517 | | |  |
| Former | 16682 | 0.06 (0.00, inf.) 0.9902 |  | 0.82 (0.66, 1.01) 0.0590 | | 0.77 (0.61, 0.97) 0.0253 | | | 1.06 (0.85, 1.34) 0.5941 | | |  |
| Never | 20387 | 0.07 (0.00, inf.) 0.9875 |  | 1.40 (1.13, 1.73) 0.0018 | | 1.34 (1.06, 1.69) 0.0127 | | | 1.15 (0.89, 1.49) 0.2737 | | |  |
| **Qualifications (%)** |  |  | 0.3575 |  | 0.7433 | |  | 0.4612 | |  | 0.0135 | |
| With college or university degree | 315886 | 0.91 (0.80, 1.02) 0.1166 | | 0.94 (0.90, 1.00) 0.0395 | | 0.89 (0.84, 0.94) <0.0001 | | | 1.06 (1.00, 1.12) 0.0502 | | |  |
| Without college or university degree | 151108 | 0.77 (0.53, 1.13) 0.1814 | | 0.93 (0.84, 1.02) 0.1271 | | 0.85 (0.77, 0.94) 0.0013 | | | 0.91 (0.83, 1.01) 0.0837 | | |  |
| **Diabetes history** |  |  | 0.0116 |  | 0.8131 | |  | 0.7493 | |  | 0.807 | |
| Yes | 24210 | 0.05 (0.00, inf.) 0.9919 |  | 0.91 (0.78, 1.05) 0.1850 | | 0.83 (0.71, 0.98) 0.0267 | | | 1.03 (0.88, 1.20) 0.7292 | | |  |
| No | 441303 | 0.84 (0.72, 0.98) 0.0271 | | 0.95 (0.90, 0.99) 0.0295 | | 0.88 (0.84, 0.93) <0.0001 | | | 1.02 (0.97, 1.08) 0.3725 | | |  |
| **Hypertension** |  |  | 0.0004 |  | 0.1345 | |  | 0.4202 | |  | 0.113 | |
| Yes | 111708 | 1.00 (0.91, 1.10) 0.9827 | | 0.90 (0.83, 0.97) 0.0053 | | 0.86 (0.79, 0.93) 0.0001 | | | 0.97 (0.90, 1.05) 0.4847 | | |  |
| No | 355286 | 0.06 (0.00, inf.) 0.9658 |  | 0.97 (0.91, 1.03) 0.2850 | | 0.89 (0.84, 0.95) 0.0003 | | | 1.06 (0.99, 1.12) 0.0963 | | |  |
| **BMI** |  |  | 0.0574 |  | 0.8487 | |  | 0.6937 | |  | 0.7496 | |
| ≤25 | 154618 | 0.06 (0.00, inf.) 0.9771 |  | 0.93 (0.84, 1.03) 0.1753 | | 0.89 (0.80, 0.98) 0.0202 | | | 1.07 (0.96, 1.20) 0.2099 | | |  |
| >25 | 312376 | 0.92 (0.82, 1.03) 0.1308 | | 0.94 (0.89, 0.99) 0.0276 | | 0.87 (0.82, 0.92) <0.0001 | | | 1.05 (1.00, 1.11) 0.0723 | | |  |

Table S4. Stratified analyses of WBC category.

|  | WBC | | | NEUT | | NEUT% | | LYMPH% |  | PDW |  | PLT |
| --- | --- | --- | --- | --- | --- | --- | --- | --- | --- | --- | --- | --- |
|  | N | HR (95% CI) P-value | p for Interaction | HR (95% CI) P-value | p for Interaction | HR (95% CI) P-value | p for Interaction | HR (95% CI) P-value | p for Interaction | HR (95% CI) P-value | p for Interaction | HR (95% CI) P-value |
| **Age** |  |  | 0.4084 |  | 0.5547 |  | 0.5547 |  | 0.8064 |  | 0.7169 |  |
| <=60 | 287423 | 1.04 (1.02, 1.05) <0.0001 |  | 1.14 (1.10, 1.18) <0.0001 |  | 1.14 (1.10, 1.18) <0.0001 |  | 1.19 (1.10, 1.28) <0.0001 |  | 0.82 (0.76, 0.89) <0.0001 |  | 1.07 (1.00, 1.15) 0.0614 |
| >60 | 179571 | 1.05 (1.02, 1.08) 0.0005 |  | 1.16 (1.11, 1.21) <0.0001 |  | 1.16 (1.11, 1.21) <0.0001 |  | 1.20 (1.13, 1.28) <0.0001 |  | 0.80 (0.76, 0.86) <0.0001 |  | 1.13 (1.06, 1.20) <0.0001 |
| **Gender** |  |  | 0.3255 |  | 0.8355 |  | 0.757 |  | 0.9461 |  | 0.7097 |  |
| Male | 214089 | 1.05 (1.03, 1.07) <0.0001 |  | 1.14 (1.09, 1.19) <0.0001 |  | 1.17 (1.10, 1.24) <0.0001 |  | 0.85 (0.80, 0.91) <0.0001 |  | 1.06 (1.00, 1.12) 0.0411 |  | 1.12 (1.06, 1.19) <0.0001 |
| Female | 252905 | 1.04 (1.02, 1.05) <0.0001 |  | 1.14 (1.10, 1.19) <0.0001 |  | 1.18 (1.09, 1.29) <0.0001 |  | 0.85 (0.79, 0.93) 0.0002 |  | 1.08 (1.00, 1.17) 0.0510 |  | 1.15 (1.07, 1.23) 0.0001 |
| **Race** |  |  | 0.1599 |  | 0.0283 |  | 0.1493 |  | 0.1905 |  | 0.0928 |  |
| White European | 441207 | 1.04 (1.03, 1.05) <0.0001 |  | 1.14 (1.10, 1.17) <0.0001 |  | 1.17 (1.11, 1.23) <0.0001 |  | 0.85 (0.81, 0.90) <0.0001 |  | 1.08 (1.03, 1.13) 0.0024 |  | 1.13 (1.08, 1.18) <0.0001 |
| Mixed | 2717 | 0.57 (0.22, 1.50) 0.2560 |  | 0.72 (0.33, 1.58) 0.4119 |  | 0.96 (0.45, 2.08) 0.9231 |  | 1.03 (0.47, 2.25) 0.9498 |  | 0.83 (0.37, 1.85) 0.6515 |  | 1.88 (0.77, 4.58) 0.1645 |
| South Asian | 8798 | 1.40 (0.91, 2.14) 0.1262 |  | 1.27 (0.83, 1.95) 0.2669 |  | 1.03 (0.67, 1.58) 0.8888 |  | 1.06 (0.70, 1.61) 0.7778 |  | 0.60 (0.38, 0.95) 0.0283 |  | 1.47 (1.11, 1.94) 0.0067 |
| Black | 7073 | 1.26 (0.70, 2.25) 0.4397 |  | 1.38 (0.84, 2.29) 0.2077 |  | 1.40 (0.88, 2.22) 0.1604 |  | 0.71 (0.44, 1.12) 0.1406 |  | 0.93 (0.56, 1.55) 0.7828 |  | 0.99 (0.55, 1.79) 0.9796 |
| Others | 7199 | 1.25 (1.06, 1.46) 0.0066 |  | 1.79 (1.38, 2.31) <0.0001 |  | 2.10 (1.29, 3.43) 0.0028 |  | 0.51 (0.31, 0.84) 0.0083 |  | 1.26 (0.82, 1.93) 0.2954 |  | 1.18 (0.72, 1.94) 0.5094 |
| **Smoking status (%)** |  |  | 0.4443 |  | 0.6661 |  | 0.4378 |  | 0.2099 |  | 0.3507 |  |
| Current | 48996 | 1.06 (1.02, 1.10) 0.0047 |  | 1.17 (1.06, 1.28) 0.0010 |  | 1.15 (1.01, 1.31) 0.0395 |  | 0.88 (0.77, 1.01) 0.0594 |  | 1.11 (0.99, 1.25) 0.0840 |  | 1.10 (0.98, 1.24) 0.0982 |
| Former | 161651 | 1.03 (1.02, 1.05) <0.0001 |  | 1.13 (1.08, 1.18) <0.0001 |  | 1.14 (1.05, 1.22) 0.0009 |  | 0.88 (0.81, 0.95) 0.0008 |  | 1.02 (0.95, 1.09) 0.6694 |  | 1.18 (1.11, 1.26) <0.0001 |
| Never | 254506 | 1.07 (1.03, 1.11) 0.0002 |  | 1.15 (1.10, 1.21) <0.0001 |  | 1.22 (1.13, 1.31) <0.0001 |  | 0.82 (0.76, 0.88) <0.0001 |  | 1.10 (1.03, 1.18) 0.0053 |  | 1.09 (1.02, 1.17) 0.0165 |
| **Alcohol drinker status (%)** |  |  | 0.0426 |  | 0.1577 |  | 0.6237 |  | 0.1708 |  | 0.276 |  |
| Current | 429274 | 1.06 (1.04, 1.08) <0.0001 |  | 1.16 (1.12, 1.21) <0.0001 |  | 1.18 (1.12, 1.24) <0.0001 |  | 0.84 (0.80, 0.89) <0.0001 |  | 1.07 (1.02, 1.12) 0.0086 |  | 1.13 (1.08, 1.19) <0.0001 |
| Former | 16682 | 1.01 (0.91, 1.12) 0.8870 |  | 1.07 (0.95, 1.20) 0.2441 |  | 1.28 (1.03, 1.59) 0.0258 |  | 0.76 (0.61, 0.95) 0.0168 |  | 1.23 (1.01, 1.50) 0.0402 |  | 1.16 (0.96, 1.40) 0.1368 |
| Never | 20387 | 1.00 (0.81, 1.24) 0.9855 |  | 1.01 (0.79, 1.30) 0.9185 |  | 1.04 (0.81, 1.32) 0.7775 |  | 1.06 (0.83, 1.35) 0.6483 |  | 0.92 (0.71, 1.18) 0.5101 |  | 1.18 (0.95, 1.47) 0.1416 |
| **Qualifications (%)** |  |  | 0.0418 |  | 0.5879 |  | 0.1631 |  | 0.1762 |  | 0.6155 |  |
| With college or university degree | 315886 | 1.06 (1.04, 1.08) <0.0001 |  | 1.15 (1.11, 1.20) <0.0001 |  | 1.15 (1.09, 1.22) <0.0001 |  | 0.87 (0.82, 0.92) <0.0001 |  | 1.06 (1.01, 1.12) 0.0287 |  | 1.15 (1.09, 1.21) <0.0001 |
| Without college or university degree | 151108 | 1.03 (1.00, 1.05) 0.0330 |  | 1.13 (1.08, 1.18) <0.0001 |  | 1.25 (1.13, 1.38) <0.0001 |  | 0.80 (0.72, 0.88) <0.0001 |  | 1.09 (0.99, 1.19) 0.0653 |  | 1.10 (1.00, 1.20) 0.0454 |
| **Diabetes history** |  |  | 0.0972 |  | 0.8997 |  | 0.492 |  | 0.3379 |  | 0.8515 |  |
| Yes | 24210 | 1.05 (0.92, 1.21) 0.4714 |  | 1.11 (0.97, 1.27) 0.1174 |  | 1.16 (0.99, 1.36) 0.0747 |  | 0.87 (0.74, 1.02) 0.0800 |  | 1.06 (0.91, 1.23) 0.4378 |  | 1.19 (1.03, 1.37) 0.0192 |
| No | 441303 | 1.04 (1.03, 1.05) <0.0001 |  | 1.14 (1.11, 1.18) <0.0001 |  | 1.18 (1.12, 1.24) <0.0001 |  | 0.84 (0.80, 0.89) <0.0001 |  | 1.07 (1.02, 1.12) 0.0066 |  | 1.12 (1.07, 1.18) <0.0001 |
| **Hypertension** |  |  | 0.1125 |  | 0.5641 |  | 0.6208 |  | 0.6904 |  | 0.3739 |  |
| Yes | 111708 | 1.08 (1.04, 1.13) 0.0005 |  | 1.16 (1.09, 1.24) <0.0001 |  | 1.16 (1.07, 1.25) 0.0003 |  | 0.86 (0.79, 0.93) 0.0002 |  | 1.04 (0.97, 1.12) 0.2937 |  | 1.18 (1.10, 1.26) <0.0001 |
| No | 355286 | 1.04 (1.02, 1.05) <0.0001 |  | 1.14 (1.10, 1.17) <0.0001 |  | 1.19 (1.11, 1.26) <0.0001 |  | 0.84 (0.79, 0.90) <0.0001 |  | 1.09 (1.02, 1.15) 0.0057 |  | 1.11 (1.04, 1.17) 0.0007 |
| **BMI** |  |  | 0.0082 |  | 0.5655 |  | 0.058 |  | 0.0182 |  | 0.0108 |  |
| <=25 | 154618 | 1.03 (1.01, 1.05) 0.0152 |  | 1.14 (1.09, 1.18) <0.0001 |  | 1.29 (1.16, 1.43) <0.0001 |  | 0.76 (0.68, 0.84) <0.0001 |  | 1.20 (1.09, 1.32) 0.0002 |  | 1.06 (0.96, 1.17) 0.2396 |
| >25 | 312376 | 1.07 (1.05, 1.09) <0.0001 |  | 1.15 (1.11, 1.20) <0.0001 |  | 1.15 (1.09, 1.21) <0.0001 |  | 0.87 (0.83, 0.92) <0.0001 |  | 1.04 (0.99, 1.10) 0.1375 |  | 1.16 (1.10, 1.22) <0.0001 |

Table S5. Stratified analyses of inflammation related index category

|  |  | SII | | PLR | | NLR | | PPN | |
| --- | --- | --- | --- | --- | --- | --- | --- | --- | --- |
|  | N | HR (95% CI) P-value | p for Interaction | HR (95% CI) P-value | p for Interaction | HR (95% CI) P-value | p for Interaction | HR (95% CI) P-value | p for Interaction |
| **Age** |  |  | 0.0768 |  | 0.1988 |  | 0.2619 |  | 0.3868 |
| <=60 | 287423 | 1.06 (1.02, 1.11) 0.0014 |  | 1.06 (1.00, 1.11) 0.0334 |  | 1.06 (1.03, 1.08) <0.0001 |  | 1.15 (1.09, 1.22) <0.0001 |  |
| >60 | 179571 | 1.02 (1.01, 1.03) 0.0017 |  | 1.02 (1.00, 1.03) 0.0095 |  | 1.04 (1.02, 1.05) <0.0001 |  | 1.12 (1.08, 1.16) <0.0001 |  |
| **Gender** |  |  | 0.0961 |  | 0.176 |  | 0.0569 |  | 0.0384 |
| Male | 214089 | 1.04 (1.02, 1.06) 0.0001 |  | 1.04 (1.01, 1.06) 0.0013 |  | 1.03 (1.01, 1.05) 0.0005 |  | 1.12 (1.08, 1.15) <0.0001 |  |
| Female | 252905 | 1.02 (1.00, 1.03) 0.0129 |  | 1.01 (1.00, 1.03) 0.0386 |  | 1.07 (1.04, 1.11) <0.0001 |  | 1.20 (1.13, 1.26) <0.0001 |  |
| **Race** |  |  | 0.558 |  | 0.5518 |  | 0.2119 |  | 0.0048 |
| White European | 441207 | 1.02 (1.01, 1.03) <0.0001 |  | 1.02 (1.01, 1.03) 0.0003 |  | 1.04 (1.03, 1.06) <0.0001 |  | 1.13 (1.10, 1.16) <0.0001 |  |
| Mixed | 2717 | 2.08 (0.88, 4.91) 0.0956 |  | 2.23 (1.04, 4.78) 0.0394 |  | 1.21 (0.45, 3.25) 0.7022 |  | 1.02 (0.47, 2.23) 0.9561 |  |
| South Asian | 8798 | 1.01 (0.91, 1.12) 0.8942 |  | 1.01 (0.91, 1.11) 0.9128 |  | 1.00 (0.87, 1.14) 1.0000 |  | 1.37 (1.01, 1.88) 0.0461 |  |
| Black | 7073 | 1.28 (0.77, 2.12) 0.3366 |  | 1.16 (0.62, 2.18) 0.6327 |  | 1.46 (0.95, 2.23) 0.0848 |  | 1.27 (0.74, 2.20) 0.3823 |  |
| Others | 7199 | 1.15 (0.82, 1.61) 0.4291 |  | 1.02 (0.56, 1.86) 0.9358 |  | 1.24 (1.03, 1.50) 0.0242 |  | 1.72 (1.33, 2.21) <0.0001 |  |
| **Smoking status (%)** |  |  | 0.0952 |  | 0.1118 |  | 0.4274 |  | 0.3055 |
| Current | 48996 | 1.05 (0.93, 1.20) 0.4335 |  | 1.03 (0.90, 1.17) 0.6951 |  | 1.07 (0.98, 1.17) 0.1153 |  | 1.15 (1.06, 1.25) 0.0005 |  |
| Former | 161651 | 1.06 (1.03, 1.09) 0.0004 |  | 1.06 (1.02, 1.10) 0.0019 |  | 1.03 (1.01, 1.06) 0.0091 |  | 1.14 (1.10, 1.19) <0.0001 |  |
| Never | 254506 | 1.02 (1.01, 1.03) 0.0042 |  | 1.02 (1.00, 1.03) 0.0167 |  | 1.04 (1.02, 1.06) <0.0001 |  | 1.12 (1.08, 1.16) <0.0001 |  |
| **Alcohol drinker status (%)** | |  | 0.7234 |  | 0.8417 |  | 0.9289 |  | 0.9847 |
| Current | 429274 | 1.02 (1.01, 1.03) <0.0001 |  | 1.02 (1.01, 1.03) 0.0005 |  | 1.04 (1.02, 1.05) <0.0001 |  | 1.13 (1.11, 1.16) <0.0001 |  |
| Former | 16682 | 1.10 (0.98, 1.24) 0.1056 |  | 1.06 (0.97, 1.16) 0.2035 |  | 1.04 (0.94, 1.16) 0.4175 |  | 1.13 (0.99, 1.30) 0.0783 |  |
| Never | 20387 | 1.01 (0.78, 1.31) 0.9498 |  | 1.01 (0.77, 1.32) 0.9509 |  | 0.96 (0.73, 1.25) 0.7409 |  | 1.09 (0.87, 1.35) 0.4548 |  |
| **Qualifications (%)** |  |  | 0.0871 |  | 0.1969 |  | 0.0645 |  | 0.3079 |
| With college or university degree | 315886 | 1.02 (1.01, 1.03) 0.0004 |  | 1.02 (1.01, 1.03) 0.0023 |  | 1.03 (1.02, 1.05) <0.0001 |  | 1.13 (1.10, 1.16) <0.0001 |  |
| Without college or university degree | 151108 | 1.07 (1.03, 1.11) 0.0013 |  | 1.06 (1.01, 1.11) 0.0221 |  | 1.09 (1.04, 1.13) <0.0001 |  | 1.18 (1.09, 1.26) <0.0001 |  |
| **Diabetes history** |  |  | 0.7811 |  | 0.6783 |  | 0.4399 |  | 0.593 |
| Yes | 24210 | 1.03 (0.99, 1.08) 0.1824 |  | 1.03 (0.98, 1.07) 0.2322 |  | 1.02 (0.98, 1.07) 0.3210 |  | 1.17 (1.04, 1.31) 0.0093 |  |
| No | 441303 | 1.02 (1.01, 1.03) <0.0001 |  | 1.02 (1.01, 1.03) 0.0006 |  | 1.04 (1.03, 1.06) <0.0001 |  | 1.13 (1.10, 1.16) <0.0001 |  |
| **Hypertension** |  |  | 0.1183 |  | 0.1623 |  | 0.5815 |  | 0.0926 |
| Yes | 111708 | 1.04 (1.02, 1.06) 0.0002 |  | 1.04 (1.02, 1.06) 0.0007 |  | 1.03 (1.01, 1.06) 0.0013 |  | 1.18 (1.12, 1.24) <0.0001 |  |
| No | 355286 | 1.02 (1.01, 1.03) 0.0044 |  | 1.01 (1.00, 1.03) 0.0317 |  | 1.04 (1.02, 1.06) <0.0001 |  | 1.12 (1.09, 1.16) <0.0001 |  |
| **BMI** |  |  | 0.1829 |  | 0.3525 |  | 0.2049 |  | 0.4363 |
| <=25 | 154618 | 1.06 (1.01, 1.11) 0.0107 |  | 1.05 (0.99, 1.12) 0.1087 |  | 1.06 (1.03, 1.09) <0.0001 |  | 1.16 (1.09, 1.25) <0.0001 |  |
| >25 | 312376 | 1.02 (1.01, 1.03) 0.0007 |  | 1.02 (1.01, 1.03) 0.0036 |  | 1.03 (1.02, 1.05) <0.0001 |  | 1.13 (1.10, 1.16) <0.0001 |  |
